# Integrative Magnetic Resonance Imaging and Metabolomic Characterization of a Glioblastoma Rat Model

**DOI:** 10.3390/brainsci14050409

**Published:** 2024-04-23

**Authors:** Nuria Arias-Ramos, Cecilia Vieira, Rocío Pérez-Carro, Pilar López-Larrubia

**Affiliations:** Instituto de Investigaciones Biomédicas Sols-Morreale, Consejo Superior de Investigaciones Científicas-Universidad Autónoma de Madrid (CSIC-UAM), 28029 Madrid, Spain; narias@iib.uam.es (N.A.-R.);

**Keywords:** glioblastoma, magnetic resonance imaging, magnetic resonance spectroscopy, HRMAS, preclinical models

## Abstract

Glioblastoma (GBM) stands as the most prevalent and lethal malignant brain tumor, characterized by its highly infiltrative nature. This study aimed to identify additional MRI and metabolomic biomarkers of GBM and its impact on healthy tissue using an advanced-stage C6 glioma rat model. Wistar rats underwent a stereotactic injection of C6 cells (GBM group, n = 10) or cell medium (sham group, n = 4). A multiparametric MRI, including anatomical T_2_W and T_1_W images, relaxometry maps (T_2_, T_2_*, and T_1_), the magnetization transfer ratio (MTR), and diffusion tensor imaging (DTI), was performed. Additionally, ex vivo magnetic resonance spectroscopy (MRS) HRMAS spectra were acquired. The MRI analysis revealed significant differences in the T_2_ maps, T_1_ maps, MTR, and mean diffusivity parameters between the GBM tumor and the rest of the studied regions, which were the contralateral areas of the GBM rats and both regions of the sham rats (the ipsilateral and contralateral). The ex vivo spectra revealed markers of neuronal loss, apoptosis, and higher glucose uptake by the tumor. Notably, the myo-inositol and phosphocholine levels were elevated in both the tumor and the contralateral regions of the GBM rats compared to the sham rats, suggesting the effects of the tumor on the healthy tissue. The MRI parameters related to inflammation, cellularity, and tissue integrity, along with MRS-detected metabolites, serve as potential biomarkers for the tumor evolution, treatment response, and impact on healthy tissue. These techniques can be potent tools for evaluating new drugs and treatment targets.

## 1. Introduction

Brain cancer is a life-threatening neurological disorder in which malignant cells grow, proliferate, and invade the cerebral structures of the host, seriously hampering an adequate brain function [1]. Glioblastoma (GBM) stands as the most common primary malignant brain tumor, accounting for approximately 50% of all primary malignant tumors. It is classified as a grade IV tumor by the World Health Organization (WHO) [2], the most aggressive subtype. It has an incidence of 3.26 cases/100,000 inhabitants per year in the United States, with a very poor prognosis: a 5-year survival rate of less than 7%, despite a therapeutic approach that includes surgical resection, immunotherapy, chemotherapy, and radiotherapy [3]. The infiltrative nature of this type of tumor, which makes its complete resection virtually impossible, implies an inevitable impact on the surrounding brain tissue and, ultimately, on the healthy brain tissue and its microenvironment [4]. Furthermore, the presence of the blood–brain barrier (BBB), which can hinder the delivery of drugs to the tumor [5], highlights the critical importance of researching new drug delivery methods and enhancing our tumor targeting capabilities. The current research and advancements in theranostic approaches and nanomedicine can significantly contribute to addressing this challenge [6,7,8].

Magnetic resonance imaging (MRI) stands out as one of the most powerful techniques for accurately studying and monitoring the progression of brain tumors and their effects on healthy tissue, as well as their response to treatment. Its main advantage lies in the fact it provides a wealth of information ranging from high-contrast, high-resolution anatomical images to metabolomic information with details on cell density, vascular supply, and hypoxia [9], among others.

Despite improvements, accurately assessing the tumor progression and the response to treatments using imaging techniques remains challenging. Traditionally, post-treatment tumor changes are evaluated based on anatomical post-contrast T_1_-weighted MRI images, where a decrease in the contrast-enhanced areas is interpreted as a reduced tumor burden. However, the interpretation of the image is not always straightforward due to post-surgical changes in the brain anatomy and radiation-induced necrotic areas. Additionally, the phenomenon of ‘pseudo-progression’ may raise doubts when interpreting the images [10]. Multiparametric MRI techniques offer a valuable alternative, allowing for a comprehensive assessment of the characteristics of the tumor and the healthy tissue. Diffusion tensor imaging (DTI) provides information on the tissue microstructure [11] and magnetic transfer (MT) imaging offers insights into cellularity [12], while the T_2_, T_2_*, and T_1_ mapping provide data on inflammation and vasogenic oedema [13], hemorrhage/neoangiogenesis and oxygen levels [14], and interstitial water content and BBB disruption [15], respectively. As a result, these approaches provide data that serve as imaging biomarkers of the disease progression following the therapeutic interventions that target the pathological features of the tumor. Indeed, multiparametric MRI has been utilized by other researchers in both clinical and preclinical studies for various purposes, including investigating the evolution of the development of the tumor [16], distinguishing between primary GBM tumors and metastases [17], and monitoring treatments for this disease [18,19].

On the other hand, metabolomics plays a key role in understanding the behavior of tumors and their microenvironment. The metabolites detected and identified by in vivo magnetic resonance spectroscopy (MRS), such as choline (Cho), lactate (Lac), lipids, N-acetylaspartic acid (NAA), and myo-inositol (mI), are well-studied biomarkers of the characteristics and disease progression of GBM [20]. Additionally, ex vivo High-Resolution Magic Angle Spinning (HRMAS) provides metabolomic information of a wider range of metabolites from unprocessed small tissue samples or biopsies [21,22].

Given the significant challenges posed by glioblastoma (GBM) and the increasing recognition of the importance of multiparametric MRI in understanding its pathophysiology, we aimed to characterize an advanced-stage GBM tumor model using in vivo multiparametric MRI evaluations and ex vivo metabolomic HRMAS MRS studies. Building upon prior studies utilizing multiparametric MRI in GBM research, our approach sought to provide a comprehensive assessment of the progression of a tumor and its microenvironment. By investigating both the tumor and the contralateral regions potentially affected by the tumor, along with the equivalent regions in sham animals, we aimed to discern the parameters that serve as biomarkers to monitor the disease progression. Furthermore, we aimed to explore the potential of our methodology in preclinical and clinical research, particularly in validating new drugs, including theranostic nanodrugs.

## 2. Materials and Methods

### 2.1. Animal Models

All experimental procedures complied with the national (R.D.53/2013) and European Community guidelines (2010/62/UE) for the care and management of experimental animals and were approved by the Ethics Committee of the Community of Madrid (PROEX 047/18; approved 2 November 2015). Male Wistar albino rats (*Rattus novergicus*) with a body weight (b.w.) of 230 ± 20 g were used. The animals were housed in cages in a light-controlled (12 h cycle of light and darkness) and temperature-controlled (22 ± 2 °C) room with access to water and food ad libitum in the IIBM animal facility (Reg. No. ES280790000188) and cared for by specialized personnel.

### 2.2. Cell Line Culture

An authenticated C6 glioma cell line obtained from the American Type Culture Collection (ATCC number: CCL-107) (Manassas, VA, USA) was used. The cells were cultured in Dulbecco’s Modified Eagle Medium (DMEM), supplemented with 10% fetal bovine serum (FBS) (Gibco^®^, Thermo Fisher Scientific, Inc., Waltham, MA, USA) and antibiotics (10% of amphotericin B, 100 UI/mL of penicillin, 0.03 mg/mL of gentamicin, and 0.1 mg/mL of streptomycin), and were kept in an incubator at 37 °C and 5% of CO_2_.

### 2.3. Surgical Procedure

The male Wistar rats (n = 14) were submitted to a surgical procedure using stereotaxic equipment (Model 900LS Small Animal Stereotaxic Instrument, Kopf Instruments^®^, Tujunga, CA, USA). Briefly, the animals were injected subcutaneously with the analgesic meloxicam (0.5 mg/kg b.w.) 30 min before the surgery. Then, the animals were anesthetized by an intraperitoneal injection of ketamine hydrochloride (75 mg/kg b.w.) and medetomidine hydrochloride (0.5 mg/kg b.w.) and placed in the stereotaxic device. Through a small burr hole, the tumor cells (10^5^/10 µL of culture medium per animal, ten GBM rats) or the culture medium alone (10 µL, four sham rats) were injected on the right caudate–putamen, based on coordinates using the bregma as a reference: 0.35 mm from it on the right lateral and 0.55 mm from it on the ventral side. Once finished, the skull hole was sealed and the skin sutured. After the surgery, atipamezol hydrochloride (5 mg/kg b.w.) was administered subcutaneously to fasten the anesthesia recovery, and meloxicam (0.5 mg/kg b.w.) was used for analgesia and administrated during the following two days.

### 2.4. Magnetic Resonance Imaging

The MRI studies were carried out on a 7 T superconductor horizontal animal MR system (Bruker Medical GmbH, Ettlingen, Germany) equipped with a ^1^H 38 mm bird cage resonator and a gradient insert of 90 mm in diameter (360 mT/m maximum strength). All data were acquired running Paravision 5.1 software (Bruker Medical GmbH^®^, Ettlingen, Germany) operating on a Linux platform.

The animals were anesthetized with 3–4% isoflurane in 100% O_2_ in an induction box, followed by the administration of 1.5–2% isoflurane through a mask during the MRI acquisitions. The rats were placed in an animal holder with a heated blanket, which maintained their body temperature at ~37 °C. The temperature and respiratory rate of the animals were monitored by a monitoring and gating system (SA Instruments, Inc., Stony Brook, NY, USA). The GBM-bearing rats were placed in the MRI system with a tail catheter to allow the intravenous (i.v) administration of the contrast agent (CA).

#### Magnetic Resonance Imaging Studies

The tumor development in the GBM-bearing rats was followed up with T_2_-weighted (T_2_W) anatomical MRI weekly after the surgery. Multiparametric MRI studies were conducted between 2 and 3 weeks post-surgery, when the tumor reached a volume ≥100 mm^3^, including T_2_W and T_1_-weighted (T_1_W) images after the i.v. administration of the CA and parametric MRI acquisitions: relaxometry (T_2_, T_2_*, and T_1_ maps), MT images, and DTI. The sham rats underwent the same multiparametric MRI studies.

- Anatomical MRI

The T_2_W images were acquired with a rapid acquisition relaxation-enhanced (RARE) sequence with the following acquisition parameters: a repetition time (TR) = 3000 ms, an echo time (TE) = 60 ms, the number of experiments (NEX) = 3, the total acquisition time (TAT) of 3 min and 36 s, and a RARE factor = 8, with 10 slices in an axial orientation with a slice thickness (ST) = 1.5 mm—covering the whole brain—a field of view (FOV) = 35 × 35 mm^2^, and a matrix = 256 × 256 pixels, corresponding to an in-plane resolution of 136.7 × 136.7 µm^2^. The T_1_W images were acquired after the i.v. administration of 0.3 M of Gd-diethylenetriaminepentaacetic acid (Magnevist^®^, Bayer, Whippany, NJ, USA) at a dose of 0.3 mmol/kg b.w. as the CA with the TR = 300 ms, TE = 10.5 ms, NEX = 3, and TAT = 2 min and 52 s. The same geometric parameters were used as in the T_2_W images.

- Parametric MRI

The MRI studies to generate the parametric images were performed in an axial orientation using five slices (with an ST = 1.5 mm) placed at the central part of the tumor in the GBM-bearing rats and in an equivalent position in the sham rats, with a FOV of 35 × 35 mm^2^ and a matrix = 128 × 128, corresponding to an in-plane resolution of 273.4 × 273.4 µm^2^/pixel.

The T_2_ maps were acquired using a multi-slice multi-echo (MSME) sequence with a TR = 5000 ms, employing 75 echoes; TE = 12–900 ms; NEX = 1; and TAT = 10 min and 40 s. The fitting curve for the calculation of T_2_ is described in Equation (1):

(1) $$S=S_{0}\cdot e^{-TE/T_{2}}$$

where S is the value of the MRI signal at a given TE and S_0_ is the value of the MR signal when TE = ∞.

The T_2_* maps were acquired using a multi-gradient echo (MGE) sequence with a TR = 543.3 ms, emplying 20 echoes; TE = 2.73–83.86 ms; flip angle = 30°; NEX = 4; and TAT = 5 min and 37 s. The fitting curve for the calculation of T_2_* is the one described in Equation (1), substituting T_2_ for T_2_*.

The T_1_ maps were acquired employing a saturation–recovery sequence with eight values of TR= 125–6000 ms, TE = 12 ms, NEX = 1, and TAT = 24 min and 55 s. The fitting curve for the calculation of T_1_ is described in Equation (2):

(2) $$S=S_{0}\cdot (1-e^{-TR/T_{1}})$$

where S is the value of the MR signal at a given TR and S_0_ is the value of the MRI signal when TR = ∞.

The magnetization transfer ratio (MTR) maps were generated by acquiring two set of images, one applying an MT pulse (MT ON) and the other without applying it (MT OFF), with a TR = 2500 ms, TE = 10 ms, NEX = 1, and TAT = 5 min and 20 s. The MT ONs comprised a train of radiofrequency pulses (N = 50) of bandwidth = 550 Hz, length = 5 ms, power = 5.5 µT, and offset = 1500 Hz. The MT effect was calculated as an MT ratio according to Equation (3):

(3) $$\%MTR=\frac{S_{0}-S_{MT}}{S_{0}}*100$$

where S_MT_ is the signal intensity of a pixel in the MT ON image and S_0_ the signal of the same pixel in the MT OFF.

The DTI studies were performed using a Stejskal–Tanner sequence with a single-shot echo-planar readout, where the TR = 3000 ms, TE = 39.3 ms, NEX = 4, diffusion gradient separation (∆) = 20 ms, and diffusion gradient duration (δ) = 4 ms, with one basal image and two b factors of 300 and 1400 s/mm^2^ applied in seven directions and a TAT = 3 min. The mean diffusivity (MD) and fractional anisotropy (FA) parameters were calculated according to Equations (4) and (5), where the corresponding eigenvalues (λ1, λ2, and λ3) were obtained by solving the tensor:

(4) $$MD=\frac{\lambda 1+\lambda 2+\lambda 2}{3}$$

(5) $$FA=\frac{\sqrt{{\left(\lambda 1-MD\right)}^{2}+{\left(\lambda 2-MD\right)}^{2}+{\left(\lambda 3-MD\right)}^{2}}}{2({\lambda 1}^{2}+{\lambda 2}^{2}+{\lambda 3}^{2})}$$

### 2.5. MRI Processing

The tumor volume development in the GBM-bearing rats was followed by using the T_2_W anatomical images and manually selecting the tumor areas employing the software ImageJ (National Institutes of Health, Bethesda, MD, USA, http://rsbweb.nih.gov/ij/) and then calculated using Equation (6), where the TA (tumor area) represents the area of the tumor in each slice in mm^2^:

(6) $$Tumorvolume\left({mm}^{3}\right)=\left[TAslice1+TAslice2+\left(\ldots \right)+TAslice10\right]\times ST$$

Color-based maps were generated pixelwise from the images by fitting the signal to the appropriate equation using home-made software developed in MatLab version R2010b (The MathWorks, Nattick, MA, USA). Two regions of interest (ROIs) were manually selected and quantified using the Image J: tumoral area and the healthy contralateral region in all tumor-containing slices in the GBM-bearing rats and in the equivalent areas in the sham group (the ipsilateral and contralateral areas). Then, the mean value of each ROI, considering all selected slices for each rat, was used for the statistical analysis.

### 2.6. Ex Vivo Magnetic Resonance Spectroscopy

Immediately following the multiparametric MRI study, the rats were sacrificed using a high-power (5 kw) focused microwave (TMW-6402 C, Muromachi Kikai Co., Ltd., Tokyo, Japan), which causes an arrest of the cerebral metabolism. Then, the brains were removed from the skull and the tumor and the contralateral regions were resected from the GBM-bearing rats and the equivalent areas from the sham animals. The samples were immediately frozen in liquid nitrogen and stored at −80 °C.

The HRMAS spectra were acquired in a 11.7 T Bruker AVANCE WB spectrometer (Bruker Medical GmbH, Ettlingen, Germany) operating at 500.13 MHz at a ^1^H frequency, equipped with a triple nuclei HRMAS probe and using the Topspin 2.1 software. Briefly, a sample (10–15 mg) was placed on a zirconium oxide rotor (4 mm o.d.) and suspended in 50 μL of D_2_O. The spectra were acquired in a probe cooled to 4 °C and spun at 5 kHz using a Carr–Purcell–Meiboom–Gill sequence with the following parameters: a water saturation pulse of 2 s, a relaxation delay of 5 s, 32 k data points, and 128 scans. Two spectra per sample were acquired, one with a total TE of 36 ms and another of 144 ms. Then, the detectable metabolites were quantified using the LCModel package (Linear Combination of Model Spectra, http://s-provencher.com/lcmodel.shtml), a prior knowledge spectral fit software. This program fits the sample spectra as a linear combination of the model spectra contained in a home-designed database of brain metabolites and taking into account the contributions for lipids and macromolecules, yielding values for the metabolite concentration and estimated standard deviation (SD) [23]. Only metabolites with an SD smaller than 20% were included in the final analysis of the data. The metabolite concentrations are presented normalized to the total creatine (PCr + Cr) content.

### 2.7. Statistical Analysis

The statistical analysis and data representation were performed using GraphPad Prism Software, version 9 (GraphPad Software, La Jolla, CA, USA). The Shapiro–Wilk test was used to assess the normality of the data. A two-way ANOVA followed by Tukey’s post hoc for multiple comparison was used for the comparison among the different groups (the GBM rats vs. the sham rats). To compare the regions within the same group (tumor/ipsilateral vs. contralateral), a paired *t*-test with a Holm–Sidak correction was performed. The data are represented by boxplots, where the horizontal bar represents the median, the ‘+’ symbol shows the mean, and the lower and upper limits of the box indicate the first and third quartile, respectively. The upper and lower whiskers extend to the most extreme data points 1.5× the interquartile range from the nearest box border (the quartile). A *p*-value < 0.05 was considered statistically significant.

## 3. Results

### 3.1. MRI Studies

**Figure 1:**
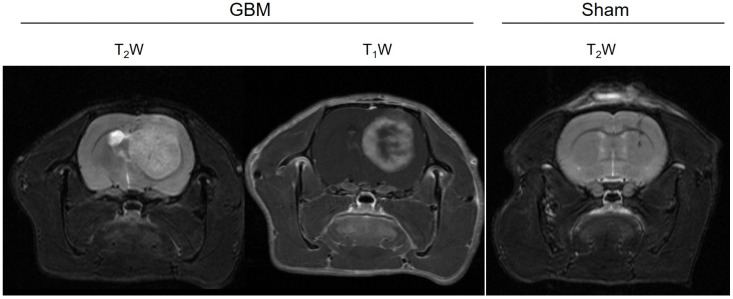
Anatomical images of a representative slice from the GBM and sham animals. The surgical scar is observed as a hypointense area in the T_2_W image of the sham rat, while the tumor is detected as an hyperintense area in the T_2_W and T_1_W images after the CA administration in the GBM rat.

The multiparametric MRI studies were conducted in the GBM rats between 16 and 21 days after the surgery, once the tumors had reached a volume of ≥100 mm^3^, while the sham rats underwent the studies 21 days after surgery. The tumors were observed in the GBM animals as hyperintense areas on both the T_2_WI (weighted images) and in the T_1_WI after the administration of the CA (Figure 1). In addition, a higher uptake of the CA could be observed in the proliferative tumor periphery region than in the central core area due to the presence of necrosis. The scars resulting from the intracranial surgery were visible as hypointense areas on the T_2_WI from the sham rats.

#### 3.1.1. Relaxometry

**Figure 2:**
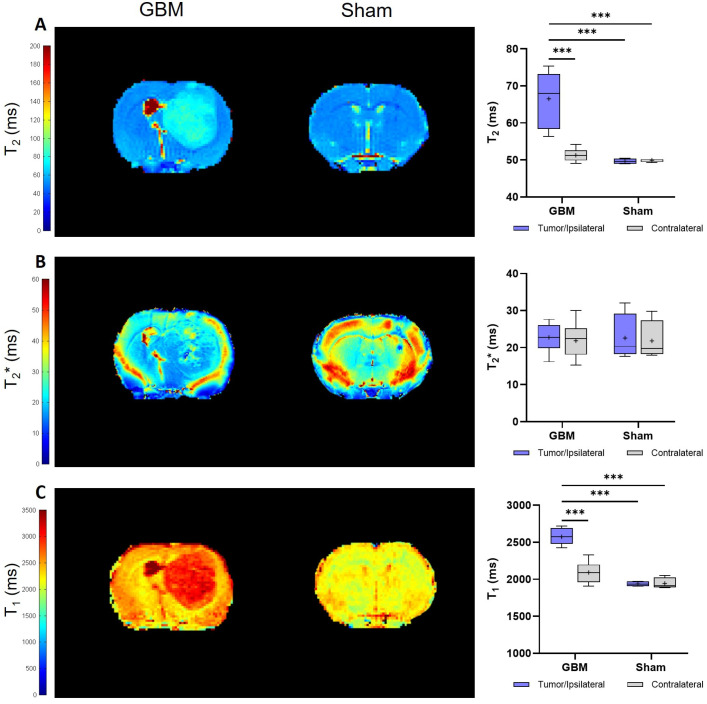
Parametric maps generated from the relaxometry images of a representative slice from the GBM and sham rats and a quantification of the studied regions: the tumor and contralateral areas in the GBM rats, as well as the ipsilateral and contralateral regions in the sham rats. (**A**). Parametric maps and quantification of the T_2_ values. (**B**). Parametric maps and quantification of the T_2_* values. (**C**). Parametric maps and quantification of the T_1_ values. *** *p* < 0.001.

The relaxation times, including the T_2_, T_2_*, and T_1_ values from the assessed regions (the tumor/ipsilateral and contralateral areas), were quantified from the corresponding parametric maps (Figure 2). The mean relaxation values for each group are presented in Table 1.

The results obtained on the T_2_ maps of this GBM model are depicted in Figure 2A. Higher T_2_ values were observed in the tumor compared to the contralateral areas in the GBM rats (*p* < 0.001) and compared to the ipsilateral and contralateral regions of the sham rats (*p* < 0.001). Similar T_2_ values were observed in the ipsilateral and contralateral regions of the sham rats.

No statistically significant differences in the T_2_* values were found between the regions in the GBM or sham animals, nor among the groups (Figure 2B).

Regarding the T_1_ values, similar results were obtained as for the T_2_ values. The tumor regions of the GBM rats showed higher T_1_ values than the corresponding contralateral areas (*p* < 0.001), and they were also higher than the ipsilateral and contralateral regions of the sham rats (*p* < 0.001). No statistically significant differences in the T_1_ values were observed between the ipsilateral and contralateral regions of the sham rats (Figure 2C).

#### 3.1.2. Magnetization Transfer Images

**Figure 3:**
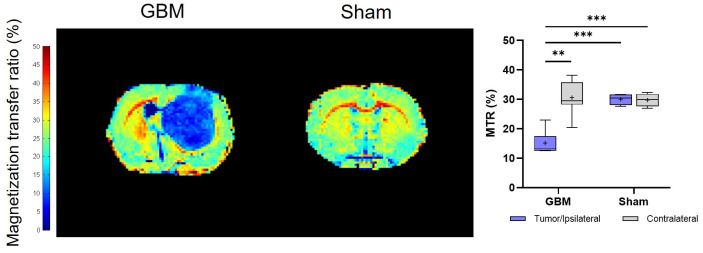
Parametric maps generated from the MT images of a representative slice from the GBM and sham rats and a quantification of the studied regions: the tumor and contralateral areas in the GBM rats, as well as the ipsilateral and contralateral regions in the sham rats. ** *p* < 0.01 and *** *p* < 0.001.

The calculated MTR values showed trends that were consistent with the T_2_ and T_1_ analyses. However, notably, lower MTR values were detected in the tumor than in the contralateral areas (*p* < 0.01) of the GBM rats, as well as in comparison to both regions studied in the sham rats (*p* < 0.001), where no significant differences were detected between regions in this group (Figure 3). The mean MTR values from each group are presented in Table 1.

#### 3.1.3. Diffusion Tensor Imaging

**Figure 4:**
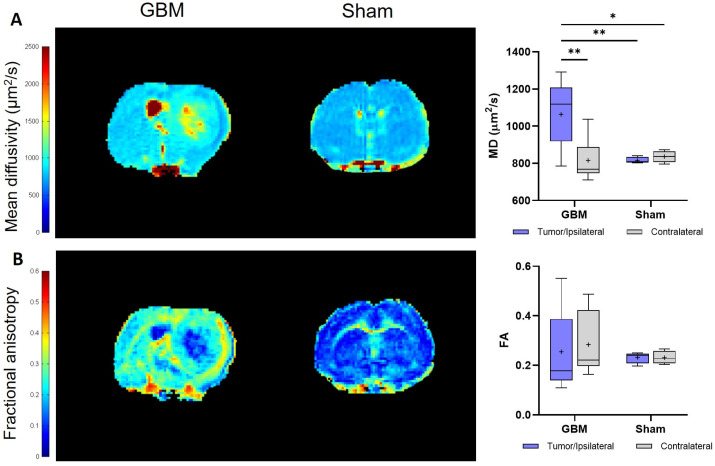
Parametric maps generated from the DTI images of a representative slice from the GBM and sham rats and a quantification of the studied regions: the tumor and contralateral areas in the GBM rats, as well as the ipsilateral and contralateral regions in the sham rats. (**A**). Parametric maps and quantification of MD values. (**B**). Parametric maps and quantification of FA values. * *p* < 0.05 and ** *p* < 0.01.

The DTI studies provide information about the restriction of the water molecule movement in the tissues and, therefore, about the tissues’ microstructural organization through the mean diffusivity (MD) and fractional anisotropy (FA) parameters, respectively. The same trend observed in the T_2_ and T_1_ analyses was observed, with the tumor regions showing higher MD values than the respective contralateral area of the GBM rats (*p* < 0.01) and, also, when compared to the ipsilateral (*p* < 0.01) and contralateral areas (*p* < 0.05) of the sham rats, with no significant differences between the regions in this last group (Figure 4A). Regarding the FA, no statistically significant differences were detected either within or among the groups. However, a greater degree of data dispersion is evident in the boxplot representation of the tumor and contralateral areas of the GBM rats compared to the regions of the sham rats (Figure 4B). The mean MD and FA values from each group are presented in Table 1.

### 3.2. Metabolomic Studies: Ex Vivo Spectra

**Figure 5:**
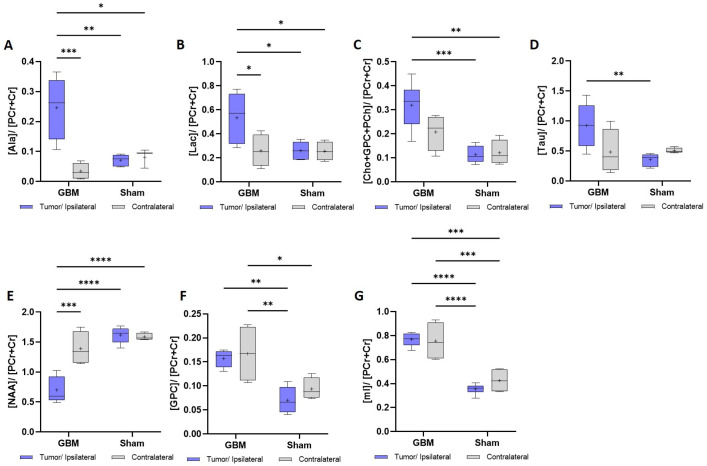
Metabolic data obtained from the ^1^H HRMAS spectra with a TE = 36 ms from the tumor and contralateral regions of the GBM rats and the ipsilateral and contralateral regions of the sham rats. The metabolic concentrations are expressed relative to the phosphocreatine + creatine (PCr + Cr). (**A**). Ala: alanine. (**B**). Lac: lactate. (**C**). Cho + GPC + PCh: choline + glycerophosphocholine + phosphocholine. (**D**). Tau: taurine. (**E**). NAA: N-acetylaspartic acid. (**F**). GPC: glycerophosphocholine. (**G**). mI: myo-inositol. * *p* < 0.05, ** *p* < 0.01, *** *p* < 0.001, and **** *p* < 0.0001.

The metabolomic information was obtained from the ^1^H HR-MAS spectra acquired from the regions of the animals across the different groups. Figure 5 shows the metabolites in which statistically significant differences were observed in the spectra acquired with a TE of 36 ms. The mean metabolite concentration data are presented in Table 2.

Higher concentrations were found in the tumor of the GBM rats when compared to the contralateral regions or when compared to the sham rats in alanine (Ala), lactate (Lac), choline + glycerophosphocholine + phosphocholine (Cho + GPC + PCh), and taurine (Tau). Similar concentrations were found when the contralateral area from the GBM rats and both regions of the sham rats were compared. The Ala and Lac exhibited statistically significant differences in the three comparisons: between the tumor and the contralateral regions in the GBM rats (*p* < 0.001), between the tumor and the sham rats (*p* < 0.01), and (*p* < 0.05) for the ipsilateral and contralateral regions in the sham animals (Figure 5A). For the Lac, all three comparisons showed significance (*p* < 0.05) (Figure 5B). A similar trend was observed for the Cho + GPC + PCh, when comparing the tumor region of the GBM rats to the ipsilateral area (*p* < 0.001) and the contralateral area (*p* < 0.01) of the sham rats. However, although there was a lower concentration in the contralateral area of the GBM rats, this difference was not statistically significant (Figure 5C). A comparable pattern to the Cho + GPC + PCh was found in the Tau, with statistically significant differences observed only between the GBM tumor and the sham ipsilateral region (*p* < 0.01) (Figure 5D).

In the case of the N-acetylaspartic acid (NAA), lower concentrations were found in the GBM tumor than in the contralateral region of the GBM rats (*p* < 0.001) and for the ipsilateral (*p* < 0.0001) and contralateral (*p* < 0.0001) sham regions (Figure 5E). In the case of the glycerophosphocholine (GPC) and myo-inositol (mI), a higher metabolite concentration was found in both the tumor and contralateral areas of the GBM rats compared to the ipsilateral and the contralateral regions of the sham rats. In the GPC, statistically significant differences were found between the tumor of the GBM rats and the ipsilateral area (*p* < 0.01) of the sham rats and, also, between the contralateral area of the GBM rats and the ipsilateral (*p* < 0.01) and contralateral areas (*p* < 0.05) of the sham rats (Figure 5F). Regarding the mI, statistically significant differences were found between the GBM tumor and the ipsilateral (*p* < 0.0001) and contralateral areas (*p* < 0.0001) of the sham rats and between the contralateral regions of the GBM rats and the ipsilateral (*p* < 0.0001) and contralateral areas of the sham rats (*p* < 0.0001) (Figure 5G).

The same trend was observed in the metabolite analysis obtained from the spectra acquired with a TE of 144 ms, shown in Figure S1 of the Supplementary Material.

## 4. Discussion

Despite advances in recent decades, GBM remains a fatal cancer with a dismal prognosis. This aggressive brain tumor presents formidable challenges in diagnosis and treatment. Efforts to improve patient outcomes hinge on the identification of precise diagnostic markers and the development of targeted therapies. The validation of these treatments is crucial for effective management. Advanced imaging modalities such as MRI and the utilization of animal models play pivotal roles in this endeavor, offering valuable insights into the tumor biology and aiding in the development and validation of novel therapeutic approaches. Furthermore, multiparametric MRI facilitates the non-invasive and quantitative assessment of multiple tissue characteristics, complementing the qualitative insights obtained from anatomical T_2_W and T_1_W images, which may also be very useful for validating theranostic approaches [8,24,25].

The aim of this study was to identify the biomarkers of GBM using in vivo MRI and ex vivo metabolomic analysis via HRMAS MRS, with the potential to enhance accurate diagnosis and an early therapy validation. Additionally, these methodologies can be applied to investigate preclinical models of GBM and to identify therapeutic targets, thereby aiding in the development of novel drugs against GBM. Moreover, beyond analyzing the tumor region, considering the infiltrative nature of GBM, our objective was to examine the apparently healthy tissue areas to ascertain potential tumor infiltration into the normal brain tissue.

Overall, we failed to detect significant signs of tumor invasion in the apparently healthy contralateral region. The most pronounced differences were observed between the tumor region of the GBM rats and the rest of the studied regions: the contralateral hemisphere of the GBM rats and the ipsilateral and contralateral regions of the sham rats. Although we were not able to detect any tumor invasion, the disparities observed within the tumor region can potentially serve as biomarkers for the detection and evolution of tumors and the therapy response. The T_2_ and T_1_ mapping showed statistically significant higher values in the tumor regions of the GBM rats. This observed increase in the T_1_ and T_2_ values can be attributed to several underlying factors. GBM typically exhibits high cellularity, increased tissue water content, and alterations in the tissue microstructure, all of which contribute to changes in the relaxation times. The higher cell density and water content in the tumor result in prolonged T_1_ and T_2_ values compared to healthy tissue. Additionally, the presence of vasogenic edema, necrotic regions, and altered vascularization within the GBM microenvironment further influences these MRI parameters, leading to an overall increase in the T_1_ and T_2_ values. Furthermore, the disruption of the BBB in the tumor contributes to the increase in the T_2_ values.

T_2_ represents the time it takes for the transverse component of the magnetization in the MRI signal to decay, and it is correlated with, among other aspects, the content of free-water in the tissue. It is indicative of the presence of vasogenic edema, a common feature in human GBM and peritumoral areas [13]. Elevated mean T_2_ values in GBM compared to normal contralateral tissues have been reported in both human and preclinical models. This can be attributed to the presence of gliosis, necrosis, and irregular vasculature within the tumor [13,26,27,28]. Moreover, decreased T_2_ values induced in the tumor and peritumoral edema have been recognized as indicators of therapy response in GBM patients undergoing antiangiogenic treatment [29,30] and radiotherapy [31]. T_1_ is the longitudinal relaxation time, corresponding to the time it takes for the longitudinal component of the magnetization to recover due to the exchange of energy between the water spins and the environment. This parameter, utilized to identify anatomical changes and BBB disruption through the extravasation of the CA, is also associated with the response of the tumor to therapy [32,33,34] and tumor infiltration [35]. While T_1_ values have not typically been viewed as potential biomarkers without the use of contrast agents, previous studies indicate that quantitative T_1_ values measured prior to injection can predict the potential of extravasation, thereby making T_1_ a potential biomarker for BBB disruption without administrating a CA [15]. In our study, we found higher T_1_ values in the tumor area than in the contralateral brain tissue and the sham rats, consistent with the existence of tumor infiltration [35], necrosis, and increased permeability of the vessels due to the BBB alteration [36,37]. This has also been observed in other preclinical models, such as neuroblastoma [38]. While perfusion evaluations were not included in our MRI protocol for this study, the data we obtained suggest that it would be beneficial to include them in future investigations.

In contrast, the obtained T_2_* values were similar among the regions. T_2_* refers to the transfer relaxation time in the presence of inhomogeneities in the magnetic field, as a result of variations in the local magnetic susceptibility. It has been reported that tumors induce the loss of brain homogeneity due to increased cellularity, aberrant microvasculature, blood accumulation from micro-hemorrhages, and edematous or necrotic areas, resulting in decreased T_2_* values in seminal pathological tissues [14]. However, this effect was not detected in our study, which warrants further investigation in future experiments.

Regarding the MTR, it is linked to the distinct distribution of water molecules between two different compartments: a free water pool (comprising water molecules with T_2_ > 10 ms) and a pool of water molecules (those with T_2_ < 1 ms) bound to macromolecules [39]. In normal physiological conditions, each type of tissue has its unique distribution of both water compartments, which may be altered in pathological situations [12]. We observed that the GBM tumor values were significantly lower than those in the contralateral hemisphere and the regions of the sham animals, consistent with prior studies using the C6 model, particularly when examining the tumor core [40]. This finding suggests the presence of necrotic areas, especially in the advanced tumor stages, as observed in our study. Furthermore, MT imaging has been identified as a biomarker of the response to therapy in human GBM, effectively distinguishing between responders and non-responder patients [41]. Interestingly, the same authors found no significant differences in this parameter between the contralateral areas of the GBM patients and healthy subjects [42], contrary to what occurs in our study.

The diffusion phenomenon is associated with the random Brownian motion of water molecules. In this study, we focused on analyzing two DTI parameters: MD and FA. The MD is influenced by various factors, including tissue organization, cell size and integrity, permeability barriers, and viscosity. Consistent with the T_2_ and T_1_ results, we observed higher MD values in the tumor region of the GBM rats compared to the other regions studied. In brain cancer, two significant factors affect the MD in opposite directions: vasogenic edema and necrosis increase the free water content, thereby elevating the MD values, while hypercellularity and cytotoxic edema decrease them [43]. Hence, our results suggest that vasogenic edema and/or necrosis in this tumor outweigh the impact of the increased cell density or cytotoxic edema, which is consistent with the results obtained from this preclinical model at an advanced stage [40,44]. The MD is associated with clinical outcomes in high-grade gliomas, serving as an indicator of changes from basal levels to post-therapeutic stages [45,46]. It is worth noting that, regarding tumor infiltration, there are some discrepancies with this parameter [47]. Furthermore, a preclinical study conducted in this C6 model, employing both fed and fasted rats, reported differences in the MD in the apparently healthy brain tissue between fasted GBM rats and fasted control rats [48].

The FA, on the other hand, reflects the degree of anisotropy in the translational movement of the water molecules and is highly dependent on the tissue composition. Structural changes should be reflected on the FA indexes and the presence of a tumor can displace normal structures and disrupt fiber tracts, thereby altering the existing preferential directionality of the water motion, reducing the FA values. It also serves as an indicator of tumor invasion [49] and has been reported to be related to therapy response [46]. In this study, we did not observe differences in the FA between the tumor area and the contralateral hemisphere or the sham regions. However, the presence of a higher data dispersion in the two studied regions of the GBM rats compared with the sham rats could be reflecting the tumor heterogeneity among animals, which also affects the microstructure of the contralateral healthy brain regions. Nevertheless, some studies have reported not only similar but even higher FA levels in the tumor in this C6 GBM model. One possible explanation is that the tumor grows in a ring-like structure, thus elevating the FA values [40,44]. Differences in the FA were also detected in several apparently healthy brain tissue areas between the GBM and control rats in both fed and fasted states [48].

In this study, we conducted an analysis of metabolomic data using magnetic resonance spectroscopy. While in vivo ^1^H MRS provides insights into tumor evolution, grading, and treatment response [50,51], a broader range of metabolites can be obtained ex vivo from biopsies using ^1^H HRMAS in both humans and preclinical models such as C6GBM [21,22,23,52].

We observed significant differences among the studied regions in several metabolites or groups of metabolites. In four of these metabolites, higher concentrations were found in the tumor region of the GBM rats compared to either the contralateral region of the GBM rats or the regions examined in the sham animals These four metabolites are recognized as GBM markers: Ala, a glucogenic amino acid which is converted to pyruvate for rapidly proliferating tumor cells [53]; Lac, a marker of anerobic metabolism visualized in necrotic tissues with anerobic metabolism in high-grade tumors [51]; Cho + GPC + PCh, a marker of increased cell turnover which can be detected within tumors [51]; and Tau, which correlates with the presence of apoptosis [54]. However, it is worth noting that, in the latter two cases, Cho + GPC + PCh and Tau, although higher values of these metabolites were detected in the GBM tumor, no significant differences were recorded between the tumor and the contralateral region of the GBM rats. The choline (Cho) levels typically exhibit higher concentrations in the center of a solid mass, decreasing towards the periphery. Studies have indicated a correlation between the tumor grade and Cho levels in astrocytomas, with higher grade tumors often showing elevated Cho concentrations. However, this association may not be present in high-grade gliomas characterized by extensive necrosis, which tend to result in a low choline peak. In such cases, increased lactate and lipid concentrations typically suppress the peaks of other metabolites, including Cho [55,56].

As anticipated, we observed a lower detection of NAA in the GBM tumor region compared to both the contralateral region of the GBM rats and the regions studied in the sham animals. NAA serves as a neuronal marker whose reduction is typically detected in pathologies such as brain cancer, which involve neural loss [51].

Regarding GPC and mI, we observed increased levels of these metabolites in the two studied regions of the GBM rats, suggesting that the apparently healthy brain tissue may be affected by the presence of the tumor, potentially indicating tumor infiltration, a phenomenon we were not able to detect via MRI. Additionally, lower metabolite detection was observed in the ipsilateral and contralateral regions of the sham animals. GPC is the most abundant phospholipid in mammalian cell membranes [57], and increased levels of GPC are considered a marker of low grade gliomas [58,59]. It is also a potential marker of the prognosis and response to treatment in GBM, when related to the PCh content [58,60,61]. Similar levels of this metabolite have been reported in C6 tumors and their contralateral areas [22], but, in this article, the tumor data were not compared with healthy or sham animals. Based on these inconsistencies, further research is needed concerning GPC in this C6 GBM model.

Finally, mI is a precursor of phosphatidylinositol, and elevated levels of mI are typically observed in well-differentiated low-grade gliomas compared to high-grade gliomas [62]. Additionally, mI is considered a marker of GBM therapy response [63]. However, contrary to our observations in this study, it has been described that the mI levels in a GBM tumor are lower than in tissue with a normal appearance [62,64]. Interestingly, other authors have reported findings similar to what we detected in this study: a non-significant increase of mI in the tissue with a normal appearance of the contralateral hemisphere in patients with untreated glioblastoma, suggesting the detection of tumor cell infiltration [63,65].

Previous studies have conducted multiparametric MRI investigations, both clinically and preclinically, to explore GBM invasion. These investigations typically involve diffusion-weighted and DTI images, perfusion-weighted imaging (PWI), FLAIR images, and contrast-enhanced T1WI, suggesting the combined use of these acquisitions as invasion biomarkers [66,67,68,69,70]. While our study expands on these approaches by incorporating additional imaging modalities, such as T_2_, T_2_*, and T_1_ parametric maps, along with magnetization transfer studies and the metabolomic assessment, future research should strive to further delineate tumor invasion in this model. Specifically, integrating specific perfusion techniques to assess the permeability of the blood–brain barrier could deepen our understanding. Furthermore, the integration of the multiparametric MRI findings with the metabolomic data obtained through HRMAS MRS offers a comprehensive approach for assessing the GBM pathophysiology. Our results suggest that the combination of imaging biomarkers, such as relaxation, diffusion, and MT mapping with metabolite concentrations, can provide valuable insights into the characteristics and behavior of tumors. By incorporating these findings into diagnostic algorithms or predictive models, clinicians may enhance diagnostic accuracy and improve the patient management strategies for GBM.

In summary, while our research offers valuable insights into the use of multiparametric MRI in characterizing GBM, we acknowledge the need for continued refinement and validation of the imaging parameters, as well as the importance of developing customized examination protocols to address the diverse research and clinical needs.

## 5. Conclusions

Despite considerable advancements, GBM remains a highly fatal cancer with a bleak prognosis. The exploration of novel therapies that target GBM is imperative given its high mortality and aggressive nature. The inherent invasiveness and the tumor behavior of GBM pose significant challenges to its treatment. This underscores the importance of investigating new treatments with animal models that might play a pivotal role, such as the one employed in this study. Moreover, the pursuit of imaging biomarkers using techniques like multiparametric MRI emerges as a critical strategy in overcoming the challenge of accurately assessing and monitoring aggressive brain tumors with a non-invasive approach.

In this work, our findings highlight the utility of multiparametric MRI in assessing various tissue characteristics, complementing the qualitative insights derived from conventional imaging techniques. Notably, our exploration of the metabolomic data revealed significant differences among the studied regions, pointing towards there being distinctive metabolic signatures in GBM. Our study revealed notable differences in the MRI parameters between the GBM tumor region and other studied areas, including the contralateral hemisphere of the GBM animals and the ipsilateral and contralateral regions from the sham animals. Despite our inability to detect tumor invasion in the contralateral area of the GBM rats, this characteristic was discernible through an ex vivo HRMAS spectroscopy. Further investigations are warranted to enhance the imaging techniques to accurately identify tumor invasion. Moving forward, the integration of multiparametric MRI and metabolomic data into diagnostic algorithms or predictive models holds promise for enhancing diagnostic accuracy and improving the patient management strategies for GBM.

## Figures and Tables

**Table 1:** MRI parameters (mean ± SEM) measured in the different regions of the studied groups.

	GBM		Sham	
MRI Parameter	Tumor	Contralateral	Ipsilateral	Contralateral
T_2_ (ms)	66.52 ± 2.43	51.26 ± 0.56	49.64 ± 0.35	49.91 ± 0.21
T_2_* (ms)	22.75 ± 1.30	21.85 ± 1.54	22.59 ± 3.23	21.81 ± 2.69
T_1_ (ms)	2574 ± 37	2090 ± 45	1937 ± 17	1942 ± 38
MTR (%)	15.15 ± 1.46	30.56 ± 1.99	30.03 ± 0.95	29.79 ± 1.10
MD (µm^2^/s)	1064 ± 52	816 ± 30	815 ± 9	836 ± 16
FA	0.255 ± 0.045	0.284 ± 0.035	0.232 ± 0.012	0.231 ± 0.013

SEM: standard error of mean, MTR: magnetization transfer ratio, MD: mean diffusivity, and FA: fractional anisotropy.

**Table 2:** Metabolite concentrations (mean ± SEM) obtained from the ex vivo ^1^H HRMAS (TE = 36 ms) spectra from the different regions of the studied groups. The metabolic concentrations are expressed relative to the phosphocreatine + creatine (PCr + Cr).

	GBM		Sham	
[Metabolite]/[PCr + Cr]	Tumor	Contralateral	Ipsilateral	Contralateral
Ala	0.25 ± 0.04	0.03 ± 0.01	0.07 ± 0.01	0.08 ± 0.02
Lac	0.53 ± 0.10	0.26 ± 0.07	0.26 ± 0.03	0.26 ± 0.04
Cho + GPC + PCh	0.32 ± 0.04	0.21 ± 0.04	0.11 ± 0.01	0.12 ± 0.03
Tau	0.93 ± 0.15	0.48 ± 0.19	0.36 ± 0.04	0.51 ± 0.02
NAA	0.70 ± 0.10	1.39 ± 0.14	1.59 ± 0.06	1.59 ± 0.03
GPC	0.16 ± 0.01	0.17 ± 0.03	0.07 ± 0.01	0.09 ± 0.01
mI	0.77 ± 0.03	0.75 ± 0.08	0.35 ± 0.02	0.43 ± 0.05

SEM: standard error of mean; TE: echo time; PCr + Cr: phosphocreatine + creatine; Ala: alanine; Lac: lactate; Cho + GPC + PCh: choline + glycerophosphocholine + phosphocholine; Tau: taurine; NAA: N-acetylaspartic acid; GPC: glycerophosphocholine; and mI: myo-inositol.

## Data Availability

The data presented in this study are available on request from the corresponding author due to the need for a formal data sharing agreement.

## Supplementary Materials

The following supporting information can be downloaded at https://www.mdpi.com/article/10.3390/brainsci14050409/s1. Figure S1: metabolic data obtained by the ^1^H HRMAS with a TE = 144 ms from the tumor and contralateral regions of the GBM rats and the ipsilateral and contralateral regions of the sham rats; and Table S1: the metabolite concentrations (mean ± SEM) obtained from the ex vivo ^1^H HRMAS (TE = 144 ms) spectra from the different regions of the studied groups.

## References

[1] Lauko A., Lo A., Ahluwalia M.S., Lathia J.D. (2022). Cancer Cell Heterogeneity & Plasticity in Glioblastoma and Brain Tumors. Semin. Cancer Biol..

[2] Louis D.N., Perry A., Wesseling P., Brat D.J., Cree I.A., Figarella-Branger D., Hawkins C., Ng H.K., Pfister S.M., Reifenberger G. (2021). The 2021 WHO Classification of Tumors of the Central Nervous System: A Summary. Neuro. Oncol..

[3] Ostrom Q.T., Price M., Neff C., Cioffi G., Waite K.A., Kruchko C., Barnholtz-Sloan J.S. (2022). CBTRUS Statistical Report: Primary Brain and Other Central Nervous System Tumors Diagnosed in the United States in 2015–2019. Neuro. Oncol..

[4] de Gooijer M.C., Guillén Navarro M., Bernards R., Wurdinger T., van Tellingen O. (2018). An Experimenter’s Guide to Glioblastoma Invasion Pathways. Trends Mol. Med..

[5] Mo F., Pellerino A., Soffietti R., Rudà R. (2021). Blood-Brain Barrier in Brain Tumors: Biology and Clinical Relevance. Int. J. Mol. Sci..

[6] Haumann R., Videira J.C., Kaspers G.J.L., van Vuurden D.G., Hulleman E. (2020). Overview of Current Drug Delivery Methods Across the Blood–Brain Barrier for the Treatment of Primary Brain Tumors. CNS Drugs.

[7] Arias-Ramos N., Ibarra L.E., Serrano-Torres M., Yagüe B., Caverzán M.D., Chesta C.A., Palacios R.E., López-Larrubia P. (2021). Iron Oxide Incorporated Conjugated Polymer Nanoparticles for Simultaneous Use in Magnetic Resonance and Fluorescent Imaging of Brain Tumors. Pharmaceutics.

[8] Suárez-García S., Arias-Ramos N., Frias C., Candiota A.P., Arús C., Lorenzo J., Ruiz-Molina D., Novio F. (2018). Dual T1/ T2 Nanoscale Coordination Polymers as Novel Contrast Agents for MRI: A Preclinical Study for Brain Tumor. ACS Appl. Mater. Interfaces.

[9] McMillan K.M., Rogers B.P., Field A.S., Laird A.R., Fine J.P., Meyerand M.E. (2006). Physiologic Characterisation of Glioblastoma Multiforme Using MRI-Based Hypoxia Mapping, Chemical Shift Imaging, Perfusion and Diffusion Maps. J. Clin. Neurosci..

[10] Abbasi A.W., Westerlaan H.E., Holtman G.A., Aden K.M., van Laar P.J., van der Hoorn A. (2018). Incidence of Tumour Progression and Pseudoprogression in High-Grade Gliomas: A Systematic Review and Meta-Analysis. Clin. Neuroradiol..

[11] Huisman T.A.G.M. (2010). Diffusion-Weighted and Diffusion Tensor Imaging of the Brain, Made Easy. Cancer Imaging.

[12] Pui M.H. (2000). Magnetization Transfer Analysis of Brain Tumor, Infection, and Infarction. J. Magn. Reson. Imaging.

[13] Oh J., Cha S., Aiken A.H., Han E.T., Crane J.C., Stainsby J.A., Wright G.A., Dillon W.P., Nelson S.J. (2005). Quantitative Apparent Diffusion Coefficients and T2 Relaxation Times in Characterizing Contrast Enhancing Brain Tumors and Regions of Peritumoral Edema. J. Magn. Reson. Imaging.

[14] Chavhan G.B., Babyn P.S., Thomas B., Shroff M.M., Mark Haacke E. (2009). Principles, Techniques, and Applications of T2*-Based MR Imaging and Its Special Applications. Radiographics.

[15] Hattingen E., Müller A., Jurcoane A., Mädler B., Ditter P., Schild H., Herrlinger U., Glas M., Kebir S. (2017). Value of Quantitative Magnetic Resonance Imaging T1-Relaxometry in Predicting Contrast-Enhancement in Glioblastoma Patients. Oncotarget.

[16] Le T.N.T., Lim H., Hamilton A.M., Parkins K.M., Chen Y., Scholl T.J., Ronald J.A. (2018). Characterization of an Orthotopic Rat Model of Glioblastoma Using Multiparametric Magnetic Resonance Imaging and Bioluminescence Imaging. Tomography.

[17] Neska-Matuszewska M., Bladowska J., Sąsiadek M., Zimny A. (2018). Differentiation of Glioblastoma Multiforme, Metastases and Primary Central Nervous System Lymphomas Using Multiparametric Perfusion and Diffusion MR Imaging of a Tumor Core and a Peritumoral Zone—Searching for a Practical Approach. PLoS ONE.

[18] Garteiser P., Doblas S., Watanabe Y., Saunders D., Hoyle J., Lerner M., He T., Floyd R.A., Towner R.A. (2010). Multiparametric Assessment of the Anti-Glioma Properties of OKN007 by Magnetic Resonance Imaging. J. Magn. Reson. Imaging.

[19] Brekke C., Williams S.C., Price J., Thorsen F., Modo M. (2007). Cellular Multiparametric MRI of Neural Stem Cell Therapy in a Rat Glioma Model. Neuroimage.

[20] Padelli F., Mazzi F., Erbetta A., Chiapparini L., Doniselli F.M., Palermo S., Aquino D., Bruzzone M.G., Cuccarini V. (2022). In Vivo Brain MR Spectroscopy in Gliomas: Clinical and Pre-Clinical Chances. Clin. Transl. Imaging.

[21] Wright A.J., Fellows G.A., Griffiths J.R., Wilson M., Bell B.A., Howe F.A. (2010). Ex-Vivo HRMAS of Adult Brain Tumours: Metabolite Quantification and Assignment of Tumour Biomarkers. Mol. Cancer.

[22] Coquery N., Stupar V., Farion R., Maunoir-Regimbal S., Barbier E.L., Rémy C., Fauvelle F. (2015). The Three Glioma Rat Models C6, F98 and RG2 Exhibit Different Metabolic Profiles: In Vivo 1H MRS and Ex Vivo 1H HRMAS Combined with Multivariate Statistics. Metabolomics.

[23] Righi V., Garciá-Martín M.L., Mucci A., Schenetti L., Tugnoli V., Lopez-Larrubia P., Cerdán S. (2018). Spatially Resolved Bioenergetic and Genetic Reprogramming Through the Brain of Rats Bearing Implanted C6 Gliomas As Detected by Multinuclear High-Resolution Magic Angle Spinning and Genomic Analysis. J. Proteome Res..

[24] Israel L.L., Galstyan A., Holler E., Ljubimova J.Y. (2020). Magnetic Iron Oxide Nanoparticles for Imaging, Targeting and Treatment of Primary and Metastatic Tumors of the Brain. J. Control. Release.

[25] Arias-Ramos N., Ferrer-Font L., Lope-Piedrafita S., Mocioiu V., Julià-Sapé M., Pumarola M., Arús C., Candiota A.P. (2017). Metabolomics of Therapy Response in Preclinical Glioblastoma: A Multi-Slice MRSI-Based Volumetric Analysis for Noninvasive Assessment of Temozolomide Treatment. Metabolites.

[26] Dortch R.D., Yankeelov T.E., Yue Z., Quarles C.C., Gore J.C., Does M.D. (2009). Evidence of Multiexponential T2 in Rat Glioblastoma. NMR Biomed..

[27] Eis M., Els T., Hoehn-Berlage M. (1995). High Resolution Quantitative Relaxation and Diffusion MRI of Three Different Experimental Brain Tumors in Rat. Magn. Reson. Med..

[28] Blasiak B., Tomanek B., Abulrob A., Iqbal U., Stanimirovic D., Albaghdadi H., Foniok T., Lun X., Forsyth P., Sutherland G.R. (2010). Detection of T2 Changes in an Early Mouse Brain Tumor. Magn. Reson. Imaging.

[29] Hattingen E., Jurcoane A., Daneshvar K., Pilatus U., Mittelbronn M., Steinbach J.P., Bähr O. (2013). Quantitative T2 Mapping of Recurrent Glioblastoma under Bevacizumab Improves Monitoring for Non-Enhancing Tumor Progression and Predicts Overall Survival. Neuro. Oncol..

[30] Lescher S., Jurcoane A., Veit A., Bähr O., Deichmann R., Hattingen E. (2015). Quantitative T1 and T2 Mapping in Recurrent Glioblastomas under Bevacizumab: Earlier Detection of Tumor Progression Compared to Conventional MRI. Neuroradiology.

[31] Tomaszewski M.R., Dominguez-Viqueira W., Ortiz A., Shi Y., Costello J.R., Enderling H., Rosenberg S.A., Gillies R.J. (2021). Heterogeneity Analysis of MRI T2 Maps for Measurement of Early Tumor Response to Radiotherapy. NMR Biomed..

[32] Kong Z., Yan C., Zhu R., Wang J., Wang Y., Wang Y., Wang R., Feng F., Ma W. (2018). Imaging Biomarkers Guided Anti-Angiogenic Therapy for Malignant Gliomas. NeuroImage Clin..

[33] Jain R. (2013). Measurements of Tumor Vascular Leakiness Using DCE in Brain Tumors: Clinical Applications. NMR Biomed..

[34] Herrmann K., Erokwu B.O., Johansen M.L., Basilion J.P., Gulani V., Griswold M.A., Flask C.A., Brady-Kalnay S.M. (2016). Dynamic Quantitative T1 Mapping in Orthotopic Brain Tumor Xenografts. Transl. Oncol..

[35] Nöth U., Tichy J., Tritt S., Bähr O., Deichmann R., Hattingen E. (2020). Quantitative T1 Mapping Indicates Tumor Infiltration beyond the Enhancing Part of Glioblastomas. NMR Biomed..

[36] Araki T., Inouye T., Suzuki H., Machida T., Iio M. (1984). Magnetic Resonance Imaging of Brain Tumors: Measurement of T1. Work in Progress. Radiology.

[37] Englund E., Brun A., Larsson E.M., Györffy-Wagner Z., Persson B. (1986). Tumours of the Central Nervous System: Proton Magnetic Resonance Relaxation Times T1 and T2 and Histopathologic Correlates. Acta Radiologica. Diagn..

[38] Zormpas-Petridis K., Poon E., Clarke M., Jerome N.P., Boult J.K.R., Blackledge M.D., Carceller F., Koers A., Barone G., Pearson A.D.J. (2020). Noninvasive MRI Native T1 Mapping Detects Response to MYCN-Targeted Therapies in the Th- MYCN Model of Neuroblastoma. Cancer Res..

[39] Henkelman R.M., Stanisz G.J., Graham S.J. (2001). Magnetization Transfer in MRI: A Review. NMR Biomed..

[40] Pérez-Carro R., Cauli O., López-Larrubia P. (2014). Multiparametric Magnetic Resonance in the Assessment of the Gender Differences in a High-Grade Glioma Rat Model. EJNMMI Res..

[41] Mehrabian H., Myrehaug S., Soliman H., Sahgal A., Stanisz G.J. (2018). Quantitative Magnetization Transfer in Monitoring Glioblastoma (GBM) Response to Therapy. Sci. Rep..

[42] Mehrabian H., Lam W.W., Myrehaug S., Sahgal A., Stanisz G.J. (2018). Glioblastoma (GBM) Effects on Quantitative MRI of Contralateral Normal Appearing White Matter. J. Neurooncol..

[43] Maier S.E., Sun Y., Mulkern R.V. (2010). Diffusion Imaging of Brain Tumors. NMR Biomed..

[44] Lope-Piedrafita S., Garcia-Martin M.L., Galons J.P., Gillies R.J., Trouard T.P. (2008). Longitudinal Diffusion Tensor Imaging in a Rat Brain Glioma Model. NMR Biomed..

[45] Chen L., Liu M., Bao J., Xia Y., Zhang J., Zhang L., Huang X., Wang J. (2013). The Correlation between Apparent Diffusion Coefficient and Tumor Cellularity in Patients: A Meta-Analysis. PLoS ONE.

[46] Mousa M.I., Youssef A., Hamed M.R., Mousa W.B., Al Ajerami Y., Akhdar H., Eisa M.H., Ibnaouf K.H., Sulieman A. (2023). Mapping High-Grade Glioma Response to Chemoradiotherapy: Insights from Fractional Anisotropy and Mean Diffusivity. J. Radiat. Res. Appl. Sci..

[47] Kinoshita M., Goto T., Okita Y., Kagawa N., Kishima H., Hashimoto N., Yoshimine T. (2010). Diffusion Tensor-Based Tumor Infiltration Index Cannot Discriminate Vasogenic Edema from Tumor-Infiltrated Edema. J. Neurooncol..

[48] Guadilla I., González S., Cerdán S., Lizarbe B., López-Larrubia P. (2023). Magnetic Resonance Imaging to Assess the Brain Response to Fasting in Glioblastoma-Bearing Rats as a Model of Cancer Anorexia. Cancer Imaging.

[49] Price S.J., Gillard J.H. (2011). Imaging Biomarkers of Brain Tumour Margin and Tumour Invasion. Br. J. Radiol..

[50] Wei L., Hong S., Yoon Y., Hwang S.N., Park J.C., Zhang Z., Olson J.J., Hu X.P., Shim H. (2012). Early Prediction of Response to Vorinostat in an Orthotopic Rat Glioma Model. NMR Biomed..

[51] Weinberg B.D., Kuruva M., Shim H., Mullins M.E. (2021). Clinical Applications of Magnetic Resonance Spectroscopy in Brain Tumors: From Diagnosis to Treatment. Radiol. Clin. N. Am..

[52] Cheng L.L., Anthony D.C., Comite A.R., Black P.M., Tzika A.A., Gonzalez R.G. (2000). Quantification of Microheterogeneity in Glioblastoma Multiforme with Ex Vivo High-Resolution Magic-Angle Spinning (HRMAS) Proton Magnetic Resonance Spectroscopy. Neuro. Oncol..

[53] Firdous S., Abid R., Nawaz Z., Bukhari F., Anwer A., Cheng L.L., Sadaf S. (2021). Dysregulated Alanine as a Potential Predictive Marker of Glioma—An Insight from Untargeted Hrmas-Nmr and Machine Learning Data. Metabolites.

[54] Opstad K.S., Bell B.A., Griffiths J.R., Howe F.A. (2009). Taurine: A Potential Marker of Apoptosis in Gliomas. Br. J. Cancer.

[55] Horská A., Barker P.B. (2010). Imaging of Brain Tumors: MR Spectroscopy and Metabolic Imaging. Neuroimaging Clin. N. Am..

[56] Farche M.K., Fachinetti N.O., da Silva L.R.P., Matos L.A., Appenzeller S., Cendes F., Reis F. (2022). Revisiting the Use of Proton Magnetic Resonance Spectroscopy in Distinguishing between Primary and Secondary Malignant Tumors of the Central Nervous System. Neuroradiol. J..

[57] Sonkar K., Ayyappan V., Tressler C.M., Adelaja O., Cai R., Cheng M., Glunde K. (2019). Focus on the Glycerophosphocholine Pathway in Choline Phospholipid Metabolism of Cancer. NMR Biomed..

[58] Righi V., Roda J.M., Paz J., Mucci A., Tugnoli V., Rodriguez-Tarduchy G., Barrios L., Schenetti L., Cerdán S., García-Martín M.L. (2009). 1H HR-MAS and Genomic Analysis of Human Tumor Biopsies Discriminate between High and Low Grade Astrocytomas. NMR Biomed..

[59] Gandía-González M.L., Cerdán S., Barrios L., López-Larrubia P., Feijoó P.G., Palpan A., Roda J.M., Solivera J. (2019). Assessment of Overall Survival in Glioma Patients as Predicted by Metabolomic Criteria. Front. Oncol..

[60] Kumar M., Arlauckas S.P., Saksena S., Verma G., Ittyerah R., Pickup S., Popov A.V., Delikatny E.J., Poptani H. (2015). Magnetic Resonance Spectroscopy for Detection of Choline Kinase Inhibition in the Treatment of Brain Tumors. Mol. Cancer Ther..

[61] Hattingen E., Bähr O., Rieger J., Blasel S., Steinbach J., Pilatus U. (2013). Phospholipid Metabolites in Recurrent Glioblastoma: In Vivo Markers Detect Different Tumor Phenotypes before and under Antiangiogenic Therapy. PLoS ONE.

[62] Castillo M., Smith J.K., Kwock L. (2000). Correlation of Myo-Inositol Levels and Grading of Cerebral Astrocytomas. AJNR Am. J. Neuroradiol..

[63] Steidl E., Pilatus U., Hattingen E., Steinbach J.P., Zanella F., Ronellenfitsch M.W., Bahr O. (2016). Myoinositol as a Biomarker in Recurrent Glioblastoma Treated with Bevacizumab: A 1H-Magnetic Resonance Spectroscopy Study. PLoS ONE.

[64] Candiota A.P., Majós C., Julià-Sapé M., Cabañas M., Acebes J.J., Moreno-Torres A., Griffiths J.R., Arús C. (2011). Non-Invasive Grading of Astrocytic Tumours from the Relative Contents of Myo-Inositol and Glycine Measured by in Vivo MRS. JBR-BTR.

[65] Kallenberg K., Bock H.C., Helms G., Jung K., Wrede A., Buhk J.H., Giese A., Frahm J., Strik H., Dechent P. (2009). Untreated Glioblastoma Multiforme: Increased Myo-Inositol and Glutamine Levels in the Contralateral Cerebral Hemisphere at Proton MR Spectroscopy. Radiology.

[66] Durst C.R., Raghavan P., Shaffrey M.E., Schiff D., Lopes M.B., Sheehan J.P., Tustison N.J., Patrie J.T., Xin W., Elias W.J. (2014). Multimodal MR Imaging Model to Predict Tumor Infiltration in Patients with Gliomas. Neuroradiology.

[67] Fathi Kazerooni A., Nabil M., Zeinali Zadeh M., Firouznia K., Azmoudeh-Ardalan F., Frangi A.F., Davatzikos C., Saligheh Rad H. (2018). Characterization of Active and Infiltrative Tumorous Subregions from Normal Tissue in Brain Gliomas Using Multiparametric MRI. J. Magn. Reson. Imaging.

[68] Oltra-Sastre M., Fuster-Garcia E., Juan-Albarracin J., Sáez C., Perez-Girbes A., Sanz-Requena R., Revert-Ventura A., Mocholi A., Urchueguia J., Hervas A. (2019). Multi-Parametric MR Imaging Biomarkers Associated to Clinical Outcomes in Gliomas: A Systematic Review. Curr. Med. Imaging Former. Curr. Med. Imaging Rev..

[69] Hu L.S., Ning S., Eschbacher J.M., Gaw N., Dueck A.C., Smith K.A., Nakaji P., Plasencia J., Ranjbar S., Price S.J. (2015). Multi-Parametric MRI and Texture Analysis to Visualize Spatial Histologic Heterogeneity and Tumor Extent in Glioblastoma. PLoS ONE.

[70] Li C., Wang S., Yan J.L., Torheim T., Boonzaier N.R., Sinha R., Matys T., Markowetz F., Price S.J. (2020). Characterizing Tumor Invasiveness of Glioblastoma Using Multiparametric Magnetic Resonance Imaging. J. Neurosurg..

