# Supplementary material for: Integrative Magnetic Resonance Imaging and Metabolomic Characterization of a Glioblastoma Rat Model
Source: Brain Sci. 2024 Apr 23;14(5):409. doi: 10.3390/brainsci14050409 (PMC11118082; doi:10.3390/brainsci14050409)
Supplement: Supplementary file 1 [file brainsci-14-00409-s001.zip › brainsci-2951747-supplementary material.pdf]

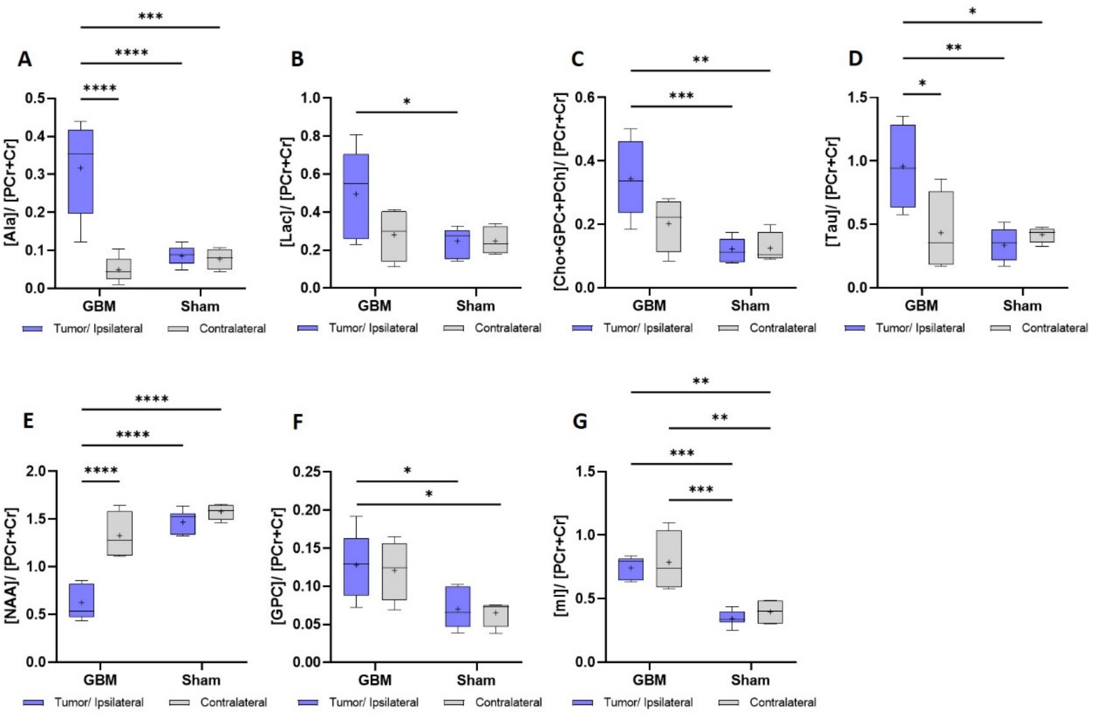

Figure S1. Metabolic data obtained from ^{1}H HRMAS spectra with a TE= 144 ms from tumor and contralateral regions of GBM rats and ipsilateral and contralateral regions of Sham rats. Metabolic concentrations are expressed relative to phosphocreatine + creatine (PCr + Cr). A. Ala: alanine. B. Lac: lactate. C. Cho+GPC+PCh: choline + glycerophosphocholine + phosphocholine. D. Tau: taurine. E. NAA: N-acetylaspartic acid. F. GPC: glycerophosphocholine, G. mI: myo-inositol. *p< 0.05, ** p< 0.01, *** p< 0.001 and **** p< 0.0001.

Table S1. Metabolite concentrations (mean ± SEM) obtained from *ex vivo* ^{1}H HRMAS (TE= 144 ms) spectra from different regions of the studied groups. Metabolic concentrations are expressed relative to phosphocreatine + creatine (PCr + Cr).

	**GBM**		**Sham**	
**[Metabolite]/ [PCr + Cr]**	**Tumor**	**Contralateral**	**Ipsilateral**	**Contralateral**
Ala	0.32 ± 0.06	0.05 ± 0.02	0.09 ± 0.01	0.08 ± 0.01
Lac	0.49 ± 0.11	0.28 ± 0.07	0.25 ± 0.03	0.25 ± 0.04
Cho+GPC+PCh	0.34 ± 0.05	0.20 ± 0.04	0.12 ± 0.01	0.12 ± 0.02
Tau	0.96 ± 0.15	0.43 ± 0.16	0.34 ± 0.05	0.42 ± 0.03
NAA	0.63 ± 0.08	1.33 ± 0.13	1.47 ± 0.05	1.58 ± 0.04
GPC	0.13 ± 0.02	0.12 ± 0.02	0.07 ± 0.01	0.06 ± 0.01
mI	0.74 ± 0.04	0.79 ± 0.12	0.34 ± 0.02	0.40 ± 0.05

SEM: standard error of mean, TE: echo time, PCr+Cr: phosphocreatine + creatine, Ala: alanine, Lac: lactate, Cho+GPC+PCh: choline + glycerophosphocholine + phosphocholine, Tau: taurine, NAA: N-acetylaspartic acid, GPC: glycerophosphocholine. mI: myo-inositol.
